# Pituitary apoplexy during pregnancy

**DOI:** 10.11604/pamj.2014.17.211.4133

**Published:** 2014-03-17

**Authors:** Hakima Chegour, Nawal El Ansari

**Affiliations:** 1 Department of Endocrinology Diabetes and Metabolic Diseases, Laboratory Pneumo cardiovascular immunopathology and Metabolism, Faculty of Medicine and Pharmacy of Marrakesh, Cadi Ayyad University, University Hospital Mohamed VI, Marrakesh, Morocco

**Keywords:** Pituitary apoplexy, pregnancy, galactorrhea, headaches

## Image in medicine

The pituitary apoplexy is a rare and fatal complication of the pituitary adenoma; it represents 0.6 to 10%. This is a clinical syndrome resulting from a fulminant pituitary expansion due to a bleeding and\or a pituitary infarcissement. We report the case of a 29-year-old patient who consults for an amenorrhoea with spontaneous galactorrhea and chronic headaches evolving over 7 months. The balance sheet of the amenorrhoea discovers a hyperprolactinemia in 224ng / ml treated by Bromocriptine without étiological survey. The patient had a pregnancy, and in 19 weeks of amenorrhoea, she presents of rough installation of the rebel headaches with visual disorders (blindness of the right eye). The magnetic resonance imaging (MRI) was in favour of a stroke of the pituitary macro-adenoma (A and B). The prolactin was always brought up. The patient was treated by Cabergoline. The evolution was favorable, with complete regression of the visual disorders after one month of treatment. The MRI of control shows the total disappearance of the leaving expansive process place in an intracellair arachnoidocèle (C and D). The clinical demonstrations of the pituitaire stroke are essentially represented by the tumoral syndrome, the visual disorders and the disorders of consciousness. The MRI is the examination of choice. In the case reported radiographic appearance was typical of a pituitary apoplexy.

**Figure 1:**
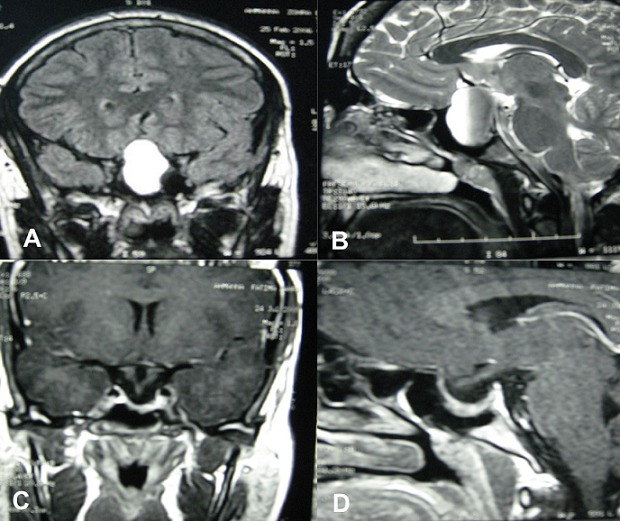
The magnetic resonance imaging (MRI) was in favour of a stroke of the pituitary macro-adenoma(A and B). The MRI of control shows the total disappearance of the leaving expansive process place in an intracellair arachnoidocèle (C and D)

